# Substantial undocumented infection facilitates the rapid dissemination of novel coronavirus (SARS-CoV-2)

**DOI:** 10.1126/science.abb3221

**Published:** 2020-03-16

**Authors:** Ruiyun Li, Sen Pei, Bin Chen, Yimeng Song, Tao Zhang, Wan Yang, Jeffrey Shaman

**Affiliations:** 1 MRC Centre for Global Infectious Disease Analysis, Department of Infectious Disease Epidemiology, School of Public Health, Faculty of Medicine, Imperial College London, London W2 1PG, UK.; 2 Department of Environmental Health Sciences, Mailman School of Public Health, Columbia University, New York, NY 10032, USA.; 3 Department of Land, Air and Water Resources, University of California, Davis, Davis, CA 95616, USA.; 4 Department of Urban Planning and Design, The University of Hong Kong, Hong Kong.; 5 Ministry of Education Key Laboratory for Earth System Modeling, Department of Earth System Science, Tsinghua University, Beijing 10084, P. R. China.; 6 Department of Epidemiology, Mailman School of Public Health, Columbia University, New York, NY 10032, USA.

## Abstract

Estimation of the prevalence and contagiousness of undocumented novel coronavirus [severe acute respiratory syndrome–coronavirus 2 (SARS-CoV-2)] infections is critical for understanding the overall prevalence and pandemic potential of this disease. Here, we use observations of reported infection within China, in conjunction with mobility data, a networked dynamic metapopulation model, and Bayesian inference, to infer critical epidemiological characteristics associated with SARS-CoV-2, including the fraction of undocumented infections and their contagiousness. We estimate that 86% of all infections were undocumented [95% credible interval (CI): 82–90%] before the 23 January 2020 travel restrictions. The transmission rate of undocumented infections per person was 55% the transmission rate of documented infections (95% CI: 46–62%), yet, because of their greater numbers, undocumented infections were the source of 79% of the documented cases. These findings explain the rapid geographic spread of SARS-CoV-2 and indicate that containment of this virus will be particularly challenging.

## Undetected cases

The virus causing coronavirus disease 2019 (COVID-19) has now become pandemic. How has it managed to spread from China to all around the world within 3 to 4 months? Li *et al.* used multiple sources to infer the proportion of early infections that went undetected and their contribution to virus spread. The researchers combined data from Tencent, one of the world's largest social media and technology companies, with a networked dynamic metapopulation model and Bayesian inference to analyze early spread within China. They estimate that ∼86% of cases were undocumented before travel restrictions were put in place. Before travel restriction and personal isolation were implemented, the transmission rate of undocumented infections was a little more than half that of the known cases. However, because of their greater numbers, undocumented infections were the source for ∼80% of the documented cases. Immediately after travel restrictions were imposed, ∼65% of cases were documented. These findings help to explain the lightning-fast spread of this virus around the world.

*Science*, this issue p. 489

## Teaser

Prior to travel restrictions, most SARS-CoV-2 infections went undocumented and substantially contributed to global virus spread.

The novel coronavirus that emerged in Wuhan, China, at the end of 2019, severe acute respiratory syndrome–coronavirus 2 (SARS-CoV-2), quickly spread to all Chinese provinces and, as of 1 March 2020, to 58 other countries (*1*, *2*). Efforts to contain the virus are ongoing; however, given the many uncertainties regarding pathogen transmissibility and virulence, the effectiveness of these efforts is unknown.

The fraction of undocumented but infectious cases is a critical epidemiological characteristic that modulates the pandemic potential of an emergent respiratory virus (*3*–*6*). These undocumented infections often go unrecognized owing to mild, limited, or lack of symptoms and thus, depending on their contagiousness and numbers, can expose a far greater portion of the population to the virus than would otherwise occur. Here, to assess the full epidemic potential of SARS-CoV-2, we use a model-inference framework to estimate the contagiousness and proportion of undocumented infections in China during the weeks before and after the shutdown of travel in and out of Wuhan.

We developed a mathematical model that simulates the spatiotemporal dynamics of infections among 375 Chinese cities (see supplementary materials). In the model, we divided infections into two classes: (i) documented infected individuals with symptoms severe enough to be confirmed, i.e., observed infections; and (ii) undocumented infected individuals. These two classes of infection have separate rates of transmission: β, the transmission rate due to documented infected individuals; and μβ, the transmission rate due to undocumented individuals, which is β reduced by a factor μ.

Spatial spread of SARS-CoV-2 across cities is captured by the daily number of people traveling from city *j* to city *i* and a multiplicative factor. Specifically, daily numbers of travelers between 375 Chinese cities during the Spring Festival period (“Chunyun”) were derived from human mobility data collected by the Tencent location-based service during the 2018 Chunyun period (1 February–12 March 2018) (*7*). Chunyun is a period of 40 days—15 days before and 25 days after the Lunar New Year—during which there are high rates of travel within China. To estimate human mobility during the 2020 Chunyun period, which began 10 January, we aligned the 2018 Tencent data on the basis of relative timing to the Spring Festival. For example, we used mobility data from 1 February 2018 to represent human movement on 10 January 2020, as these days were similarly distant from the Lunar New Year. During the 2018 Chunyun, 1.73 billion travel events were captured in the Tencent data, whereas 2.97 billion trips were reported by the Ministry of Transport of the People’s Republic of China (*7*). To compensate for underreporting and reconcile these two numbers, a travel multiplicative factor, θ, which is greater than 1, is included (see supplementary materials).

To infer SARS-CoV-2 transmission dynamics during the early stage of the outbreak, we simulated observations during 10–23 January 2020 (i.e., the period before the initiation of travel restrictions) (fig. S1) using an iterated filter-ensemble adjustment Kalman filter framework (*8*–*10*). With this combined model-inference system, we estimated the trajectories of four model state variables (*S_i_*, *E_i_*, $I_{i}^{r}$, and $I_{i}^{u}$: the susceptible, exposed, documented infected, and undocumented infected subpopulations in city *i*, respectively) for each of the 375 cities, while simultaneously inferring six model parameters (*Z*, *D*, μ, β, α, and θ: the average latency period, the average duration of infection, the transmission reduction factor for undocumented infections, the transmission rate for documented infections, the fraction of documented infections, and the travel multiplicative factor, respectively).

Details of model initialization, including the initial seeding of exposed and undocumented infections, are provided in the supplementary materials. To account for delays in infection confirmation, we also defined a time-to-event observation model using a gamma distribution (see supplementary materials). Specifically, for each new case in group $I_{i}^{r}$, a reporting delay *t*_d_ (in days) was generated from a gamma distribution with a mean value of *T*_d_. In fitting both synthetic and the observed outbreaks, we performed simulations with the model-inference system using different fixed values of *T*_d_ (6 days ≤ *T*_d_ ≤ 10 days) and different maximum seeding, *Seed*_max_ (1500 ≤ *Seed*_max_ ≤ 2500) (see supplementary materials) (fig. S2). The best-fitting model-inference posterior was identified by log likelihood.

## Validation of the model-inference framework

We first tested the model-inference framework versus alternate model forms and using synthetic outbreaks generated by the model in free simulation. These tests verified the ability of the model-inference framework to accurately estimate all six target model parameters simultaneously (see supplementary methods and figs. S3 to S14). The system could identify a variety of parameter combinations and distinguish outbreaks generated with high α and low μ from those generated with low α and high μ. This parameter identifiability is facilitated by the assimilation of observed case data from multiple (375) cities into the model-inference system and the incorporation of human movement into the mathematical model structure (see supplementary methods and figs. S15 and S16).

## Epidemiological characteristics during 10–23 January 2020

**Fig. 1:**
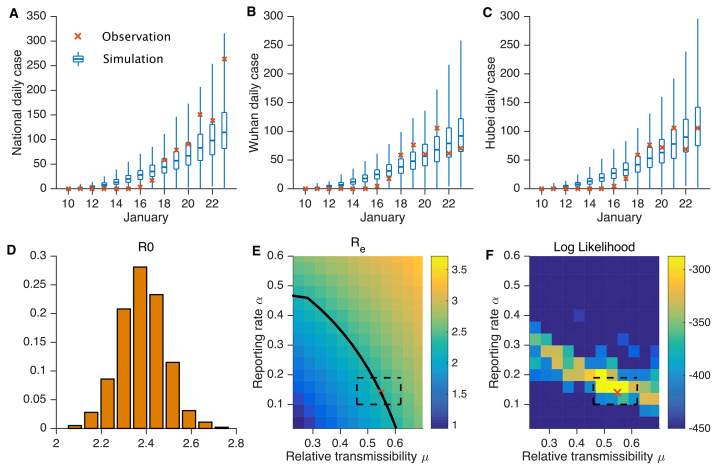
Best-fit model and sensitivity analysis. Simulation of daily reported cases in all cities (**A**), Wuhan city (**B**), and Hubei province (**C**). The blue box and whiskers show the median, interquartile range, and 95% CIs derived from 300 simulations using the best-fit model (Table 1). The red x’s are daily reported cases. (**D**) The distribution of estimated *R*_e_. (**E**) The impact of varying α and μ on *R*_e_ with all other parameters held constant at Table 1 mean values. The black solid line indicates parameter combinations of (α,μ) yielding *R*_e_ = 2.38. The estimated parameter combination α = 0.14 and μ = 0.55 is indicated by the red x; the dashed box indicates the 95% credible interval of that estimate. (**F**) Log likelihood for simulations with combinations of (α,μ) and all other parameters held constant at Table 1 mean values. For each parameter combination, 300 simulations were performed. The best-fit estimated parameter combination α = 0.14 and μ = 0.55 is indicated by the red x (the x is plotted at the lower-left corner of its respective heat map pixel, i.e., the pixel with the highest log likelihood); the dashed box indicates the 95% CI of that estimate.

We next applied the model-inference framework to the observed outbreak before the travel restrictions imposed on 23 January 2020—a total of 801 documented cases throughout China, as reported by 8 February (*1*). Figure 1, A to C, shows simulations of reported cases generated using the best-fitting model parameter estimates. The distribution of these stochastic simulations captures the range of observed cases well. In addition, the best-fitting model captures the spread of infections with the novel coronavirus disease 2019 (COVID-19) to other cities in China (fig. S17). Our median estimate of the effective reproductive number, *R*_e_—equivalent to the basic reproductive number, *R*_0_, at the beginning of the epidemic—is 2.38 [95% credible interval (CI): 2.03−2.77], indicating that COVID-19 has a high capacity for sustained transmission (Table 1 and Fig. 1D). This finding aligns with other recent estimates of the reproductive number for this time period (*6*, *11*–*15*). In addition, the median estimates for the latency and infectious periods are ~3.69 and 3.47 days, respectively. We also find that, during 10–23 January, only 14% (95% CI: 10–18%) of total infections in China were reported. This estimate reveals a very high rate of undocumented infections: 86%. This finding is independently corroborated by the infection rate among foreign nationals evacuated from Wuhan (see supplementary materials). These undocumented infections are estimated to have been half as contagious per individual as reported infections (μ = 0.55; 95% CI: 0.46–0.62). Other model fittings made using alternate values of *T*_d_ and *Seed*_max_ or different distributional assumptions produced similar parameter estimates (figs. S18 to S22), as did estimations made using an alternate model structure with separate average infectious periods for undocumented and documented infections (see supplementary methods, table S1). Further sensitivity testing indicated that α and μ are uniquely identifiable given the model structure and abundance of observations used (see supplementary methods and Fig. 1, E and F). In particular, Fig. 1F shows that the highest log-likelihood fittings are centered in the 95% CI estimates for α and μ and drop off with distance from the best-fitting solution (α = 0.14 and μ = 0.55).

**Table 1:** Best-fit model posterior estimates of key epidemiological parameters for simulation with the full metapopulation model during 10–23 January 2020. *Seed*_max_ = 2000, *T*_d_ = 9 days.

**Parameter**	**Median (95% CIs)**
Transmission rate (β, days^−1^)	1.12 (1.06, 1.19)
Relative transmission rate (μ)	0.55 (0.46, 0.62)
Latency period (*Z*, days)	3.69 (3.30, 3.96)
Infectious period (*D*, days)	3.47 (3.15, 3.73)
Reporting rate (α)	0.14 (0.10, 0.18)
Basic reproductive number (*R*_e_)	2.38 (2.03, 2.77)
Mobility factor (θ)	1.36 (1.27, 1.45)

**Fig. 2:**
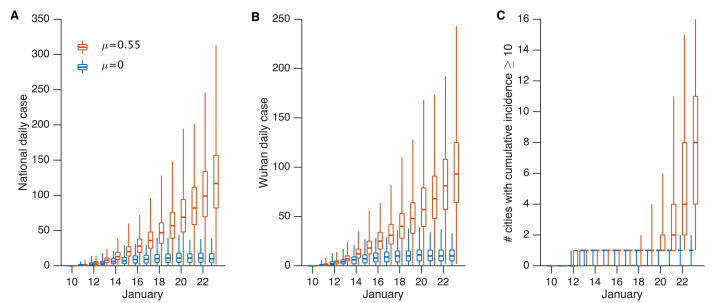
Impact of undocumented infections on the transmission of SARS-CoV-2. Simulations generated using the parameters reported in Table 1 with μ = 0.55 (red) and μ = 0 (blue) showing daily documented cases in all cities (**A**), daily documented cases in Wuhan city (**B**), and the number of cities with ≥10 cumulative documented cases (**C**). The box and whiskers show the median, interquartile range, and 95% CIs derived from 300 simulations.

Using the best-fitting model (Table 1 and Fig. 1), we estimated 13,118 (95% CI: 2974–23,435) new COVID-19 infections (documented and undocumented combined) during 10–23 January in Wuhan city. Further, 86.2% (95% CI: 81.5–89.8%) of all infections originated from undocumented cases. Nationwide, the number of infections during 10–23 January was 16,829 (95% CI: 3797–30,271), with 86.2% (95% CI: 81.6–89.8%) originating from undocumented cases. To further examine the impact of contagious, undocumented COVID-19 infections on overall transmission and reported case counts, we generated a set of hypothetical outbreaks using the best-fitting parameter estimates but with μ = 0, i.e., the undocumented infections are no longer contagious (Fig. 2). We find that without transmission from undocumented cases, reported infections during 10–23 January are reduced by 78.8% across all of China and by 66.1% in Wuhan. Further, there are fewer cities with more than 10 cumulative documented cases: only one city with more than 10 documented cases versus the 10 observed by 23 January (Fig. 2C). This finding indicates that contagious, undocumented infections facilitated the geographic spread of SARS-CoV-2 within China.

## Epidemiological characteristics after 23 January 2020

We also modeled the transmission of COVID-19 in China after 23 January, when greater control measures were effected. These control measures included travel restrictions imposed between major cities and Wuhan, self-quarantine and contact precautions advocated by the government, and more available rapid testing for infection confirmation (*11*, *12*). These measures, along with changes in medical care–seeking behavior due to increased awareness of the virus and increased personal protective behavior (e.g., wearing of face masks, social distancing, self-isolation when sick), likely altered the epidemiological characteristics of the outbreak after 23 January. To quantify these differences, we reestimated the system parameters using the model-inference framework and city-level daily cases reported between 24 January and 8 February. Given that intercity mobility was restricted after 23 January, we tested two altered travel scenarios: (i) scenario 1: a 98% reduction of travel in and out of Wuhan and an 80% reduction in travel between all other cities, as indicated by changes in the Baidu mobility index (*16*) (table S2); and (ii) scenario 2: a complete stoppage of intercity travel (i.e., θ to 0) (see supplementary methods for more details).

The results of inference for the 24 January–8 February period are presented in Table 2, figs. S23 to S26, and table S3. As control measures have continually shifted, we present estimates for both 24 January–3 February (period 1) and 24 January–8 February (period 2). For both periods, the best-fitting model for scenario 1 had a reduced reporting delay, *T*_d_, of 6 days (versus 9 days before 23 January), consistent with more rapid confirmation of infections. Estimates of both the latency and infectious periods were similar to those made for 10–23 January; however, α, β, and *R*_e_ all shifted considerably. The transmission rate of documented cases, β, dropped to 0.52 (95% CI: 0.42–0.72) during period 1 and to 0.35 (95% CI: 0.28–0.45) during period 2, less than half the estimated transmission rate prior to travel restrictions (Table 2). The fraction of all infections that were documented, α, was estimated to be 0.65 (95% CI: 0.60–0.69), i.e., 65% of infections were documented during period 1, up from 14% before travel restrictions, and remained nearly the same for period 2. The reproductive number was 1.34 (95% CI: 1.10–1.67) during period 1 and 0.98 (95% CI: 0.83–1.16) during period 2, down from 2.38 prior to travel restrictions. While the estimate for the relative transmission rate, μ, is lower than before 23 January, the contagiousness of undocumented infections, represented by μβ, was substantially reduced, possibly reflecting that only very mild, less contagious infections remain undocumented or that individual protective behavior and contact precautions have proven effective. Similar parameter estimates are derived under scenario 2 (no travel at all) (table S3). These inference results for both periods 1 and 2 should be interpreted with caution, as care-seeking behavior and control measures were continually in flux at these times.

**Table 2:** Best-fit model posterior estimates of key epidemiological parameters for simulation of the model during 24 January–3 February and 24 January–8 February. *Seed*_max_ = 2000 on 10 January, *T*_d_ = 9 days before 24 January, and *T*_d_ = 6 days between 24 January and 8 February. Travel to and from Wuhan is reduced by 98%, and other intercity travel is reduced by 80%.

**Parameter**	**24 January–3 February** **[Median (95% CIs)]**	**24 January–8 February** **[Median (95% CIs)]**
Transmission rate (β, days^−1^)	0.52 (0.42, 0.72)	0.35 (0.28, 0.45)
Relative transmission rate (μ)	0.50 (0.37, 0.69)	0.43 (0.31, 0.61)
Latency period (*Z*, days)	3.60 (3.41, 3.84)	3.42 (3.30, 3.65)
Infectious period (*D*, days)	3.14 (2.71, 3.72)	3.31 (2.96, 3.88)
Reporting rate (α)	0.65 (0.60, 0.69)	0.69 (0.65, 0.72)
Effective reproductive number (*R*_e_)	1.34 (1.10, 1.67)	0.98 (0.83, 1.16)

## Outlook

Overall, our findings indicate that a large proportion of COVID-19 infections were undocumented prior to the implementation of travel restrictions and other heightened control measures in China on 23 January and that a large proportion of the total force of infection was mediated through these undocumented infections (Table 1). This high proportion of undocumented infections, many of which were likely not severely symptomatic, appears to have facilitated the rapid spread of the virus throughout China. Indeed, suppression of the infectiousness of these undocumented cases in model simulations reduces the total number of documented cases and the overall spread of SARS-CoV-2 (Fig. 2). In addition, the best-fitting model has a reporting delay of 9 days from initial infectiousness to confirmation; in contrast, line-list data for the same 10–23 January period indicates an average 6.6-day delay from initial manifestation of symptoms to confirmation (*17*). This discrepancy suggests that presymptomatic shedding may be typical among documented infections. The relative timing of onset and peak of viremia and shedding versus onset and peak of symptoms has been shown to potentially affect outbreak control success (*18*).

Our findings also indicate that a radical increase in the identification and isolation of currently undocumented infections would be needed to fully control SARS-CoV-2. Increased news coverage and awareness of the virus in the general population have likely already prompted increased rates of seeking medical care for respiratory symptoms. In addition, awareness among health care providers and public health officials and the availability of viral identification assays suggest that capacity for identifying previously missed infections has increased. Further, general population and government response efforts have increased the use of face masks, restricted travel, delayed school reopening, and isolated suspected persons, all of which could additionally slow the spread of SARS-CoV-2.

Combined, these measures are expected to increase reporting rates, reduce the proportion of undocumented infections, and decrease the growth and spread of infection. Indeed, estimation of the epidemiological characteristics of the outbreak after 23 January in China indicates that government control efforts and population awareness have reduced the rate of virus spread (i.e., lower β, μβ, *R*_e_), increased the reporting rate, and lessened the burden on already overextended health care systems.

The situation on the ground in China is changing day to day. New travel restrictions and control measures are being imposed on populations in different cities, and these rapidly varying effects make certain estimation of the epidemiological characteristics for the outbreak difficult. Further, reporting inaccuracies and changing care-seeking behavior add another level of uncertainty to our estimations. Although the data and findings presented here indicate that travel restrictions and control measures have reduced SARS-CoV-2 transmission considerably, whether these controls are sufficient for reducing *R*_e_ below 1 for the length of time needed to eliminate the disease locally and prevent a rebound outbreak once control measures are relaxed is unclear. Moreover, similar control measures and travel restrictions would have to be implemented outside China to prevent reintroduction of the virus.

The results for 10–23 January 2020 delineate the characteristics of SARS-CoV-2 moving through a developed country, China, without major restrictions or control. These findings provide a baseline assessment of the fraction of undocumented infections and their relative infectiousness for such an environment. However, differences in control activity, viral surveillance and testing, and case definition and reporting would likely affect rates of infection documentation. Thus, the key findings, that 86% of infections went undocumented and that, per person, these undocumented infections were 55% as contagious as documented infections, could shift in other countries with different control, surveillance, and reporting practices.

Our findings underscore the seriousness of SARS-CoV-2. The 2009 H1N1 pandemic influenza virus also caused many mild cases, quickly spread globally, and eventually became endemic. Presently, there are four endemic coronavirus strains circulating in human populations (229E, HKU1, NL63, and OC43). If the novel coronavirus follows the pattern of 2009 H1N1 pandemic influenza, it will also spread globally and become a fifth endemic coronavirus within the human population.

## Supplementary Materials

science.sciencemag.org/content/368/6490/489/suppl/DC1 Materials and Methods Figs. S1 to S26 Tables S1 to S3 References (*20*–*37*) MDAR Reproducibility Checklist Data S1
